# dbSNO 2.0: a resource for exploring structural environment, functional and disease association and regulatory network of protein *S*-nitrosylation

**DOI:** 10.1093/nar/gku1176

**Published:** 2014-11-15

**Authors:** Yi-Ju Chen, Cheng-Tsung Lu, Min-Gang Su, Kai-Yao Huang, Wei-Chieh Ching, Hsiao-Hsiang Yang, Yen-Chen Liao, Yu-Ju Chen, Tzong-Yi Lee

**Affiliations:** 1 Institute of Chemistry, Academia Sinica, Taipei 115, Taiwan; 2 Department of Computer Science and Engineering, Yuan Ze University, Taoyuan 320, Taiwan; 3 Graduate Institute of Life Sciences, National Defense Medical Center, Taipei 114, Taiwan; 4 Department of Chemistry, National Taiwan University, Taipei 114, Taiwan; 5 Innovation Center for Big Data and Digital Convergence, Yuan Ze University, Taoyuan 320, Taiwan

## Abstract

Given the increasing number of proteins reported to be regulated by *S*-nitrosylation (SNO), it is considered to act, in a manner analogous to phosphorylation, as a pleiotropic regulator that elicits dual effects to regulate diverse pathophysiological processes by altering protein function, stability, and conformation change in various cancers and human disorders. Due to its importance in regulating protein functions and cell signaling, dbSNO (http://dbSNO.mbc.nctu.edu.tw) is extended as a resource for exploring structural environment of SNO substrate sites and regulatory networks of *S*-nitrosylated proteins. An increasing interest in the structural environment of PTM substrate sites motivated us to map all manually curated SNO peptides (4165 SNO sites within 2277 proteins) to PDB protein entries by sequence identity, which provides the information of spatial amino acid composition, solvent-accessible surface area, spatially neighboring amino acids, and side chain orientation for 298 substrate cysteine residues. Additionally, the annotations of protein molecular functions, biological processes, functional domains and human diseases are integrated to explore the functional and disease associations for *S*-nitrosoproteome. In this update, users are allowed to search a group of interested proteins/genes and the system reconstructs the SNO regulatory network based on the information of metabolic pathways and protein-protein interactions. Most importantly, an endogenous yet pathophysiological *S*-nitrosoproteomic dataset from colorectal cancer patients was adopted to demonstrate that dbSNO could discover potential SNO proteins involving in the regulation of NO signaling for cancer pathways.

## INTRODUCTION

Protein *S*-nitrosylation (SNO), one of the various post-translational modifications (PTMs), is reversible and involves the covalent attachment of nitric oxide (NO) to the thiol group of cysteine (Cys) residues. SNO is considered to act, in a manner analogous to phosphorylation, as a pleiotropic regulator that elicits dual effects to regulate diverse pathophysiological processes in plant (1) and human diseases, especially in cardiovascular disease and protection (2), neurodegenerative disease (3), the immune response (4), inflammation (5) and cancers (6). The various targets of SNO and differential expression of those targets modulate the activity, localization and stability of proteins (7–9). The SNO status of proteins may be linked to many cancer therapy outcomes as well as therapeutic-resistance, generating the need to develop SNO-related anti-cancer therapeutics (10). With the importance of SNO in molecular processes, numerous efforts have been directed toward mass spectrometry (MS)-based *S*-nitrosyl-proteomic studies using various biological systems to increase significantly the number of known *S*-nitrosylated peptides (11–14). Among the numerous studies, the biotin-switch method constituted a breakdown in the site-specific identification of protein SNO (15). Due to an increasing number of site-specific PTM peptides via high-throughput MS-based proteomics, a variety of PTM databases, such as dbPTM (16,17), Phospho.ELM (18), PhosphoSitePlus (19), RegPhos (20,21), O-GLYCBASE (22), dbOGAP (23), CPLM (24) and topPTM (25), have been developed. As the number of site-specific SNO peptides grows, a structured database, dbSNO (26), was developed to accumulate the experimentally verified SNO peptides by manually reviewing the research articles.

In this update, dbSNO is extended as a resource for exploring structural environment of SNO sites as well as functional and disease associations of SNO proteins. Recently, the stably growing *in vivo* or *in vitro* SNO sites have prompted an increasing interest in the structural characterization of SNO substrate sites. In this investigation, all experimentally confirmed SNO peptides were used to investigate their spatial context such as spatial amino acid composition, solvent-accessible surface area, secondary structures, side chain orientation and structurally neighboring amino acids of SNO sites. The biological roles of SNO proteins in the development and progression of cancers and diseases are varied, thus inspiring a critical need to a full investigation of disease associations for SNO proteins. Additionally, the study of *S*-nitrosoproteomics has been fueled by advances in MS-based proteomic technologies that provide researchers with the improved dbSNO for discovering NO signaling dynamics. An endogenous yet pathophysiological *S*-nitrosoproteomic data set from colorectal cancer (CRC) patients (27) was used to demonstrate that dbSNO could provide the NO regulatory network for human diseases.

## IMPROVEMENTS

The remarkable improvements and advances in dbSNO 2.0 are presented in Supplementary Figure S1, including data update for *in vivo* and *in vitro* SNO sites, structural characterization of SNO sites, functional and disease associations of SNO proteins, as well as the reconstruction of protein SNO (NO signaling) regulatory networks. To facilitate the study of protein SNO and their functions, the web interface is redesigned and enhanced. Published literature information related to SNO sites, structural characterization of SNO substrate sites, protein–protein interactions (PPIs) and metabolic pathways of SNO proteins are also provided in this online resource. The details of each improvement are depicted as follows.

### Data update for *in vivo* and *in vitro* SNO sites

All the experimentally verified SNO peptides were manually extracted from research articles by querying the keywords ‘S-nitrosylation’ or ‘*S*-nitrosylated’ against all fields of PubMed literature database. Due to a variety of proteomic identification experiments, a text-mining method is developed to extract the full-text literature that potentially describes the site-specific identification of SNO sites. Then, the full-length articles are manually reviewed for the extraction of the SNO peptides as well as the substrate cysteines. In this update, the SNO targets and sites are further defined as *in vivo* or *in vitro* according to their experimental model. For *in vivo* SNO, endogenous yet basal level, site-directed mutation on cysteine residue, induction or under stress and NO donor treatment in cultured cell is individually noted. In order to determine the precise locations of SNO cysteines within a full-length protein sequence, all *in vivo* or *in vitro* SNO peptides are mapped to UniProtKB (28) protein entries based on database identifier (ID) and sequence identity. Each mapped SNO site is attributed with a corresponding literature (PubMed ID). If the SNO sites on protein cannot assign to the same number from UniProtKB and corresponding literature, the number of SNO sites from the literature is additionally annotated to provide the experimental evidence.

### Data integration for structural characterization of SNO sites

During recent years, the steadily growing experimentally confirmed PTM sites via high-throughput MS-based proteomic techniques have prompted an increasing interest in the structural features of protein modification sites (29,30). In this update, the X-ray crystal protein structures with experimental resolution ≤2.5 Å were integrated in order to study the spatial context of SNO substrate sites. With the limit of protein structures involving the covalent attachment of NO to the thiol group of cysteine (Cys) residues, all of the experimentally verified SNO sites are mapped to the protein entries of Protein Data Bank (PDB) (31) by using Basic Local Alignment Search Tool (BLAST) with 100% sequence identity. It resulted in a total of 298 SNO sites mapping to the protein 3D structures. DSSP (32) is then utilized to calculate the surface solvent accessibility and standardize the secondary structure of PDB entries with the mapped SNO substrate sites. In the investigation of substrate site specificity, the sequential amino acid composition surrounding all SNO sites indicates no significant substrate motifs (33). Due to the difficulty of exploring the substrate motif based on linear sequences, this update investigates the propensities for the different amino acid types to occur in the spatial vicinity of the SNO substrate sites. A spatial amino acid composition (spatial AAC) is determined for all 298 mapped SNO sites by calculating the relative frequencies of 20 amino acid types within radial distances ranging from 4 to 12 Å from central SNO cysteine residues. A radial cumulative propensity plot (34) was applied to display the spatial AAC. Additionally, the side chain orientations of the amino acids spatially surrounding the SNO substrate sites are determined for investigating the functional roles and binding effects of the spatially neighboring amino acids to the substrate sites of NO attachment. As illustrated in Supplementary Figure S2, given a spatially neighboring amino acid *k*, the angle *θ_k_* less than 80° is defined as a functional residue to the cysteine residue of the SNO substrate site (35). In order to facilitate the structural investigation of protein SNO sites, all the structural characteristics were graphically presented by Jmol program (36).

### Data integration for functional and disease associations of *S*-nitrosylated proteins

To investigate the preference of biological functions for SNO proteins, dbSNO referred to the annotations of molecular function, biological process and cellular component in Gene Ontology (GO) (37). InterPro (38) is an integrated resource for providing protein ‘signatures’ such as protein families, domains and functional sites. It has been reported that the redox state and chemistry of NO facilitate its interaction with various proteins thus regulating various intracellular and intercellular events (39). Thus, the information of functional domains could be utilized to infer the functional roles of SNO sites located in a specific protein domain. During recent years, protein S-nitrosylation has been an emerging role for the development and progression of diseases and disorders, and has been developed as a therapeutic agent to reduce disease progression (39,40). Contemporary research has implicated dysregulation of SNO in severe pathological events, including cancer, disease and treatment resistance (10), motivating a necessity for a thorough investigation of *S*-nitrosoproteome dynamics. Accordingly, the disease annotations from KEGG Disease Database (41), Online Mendelian Inheritance in Man (OMIM) (42) and Human Protein Reference Database (HPRD) (43) were integrated to provide the disease associations for SNO proteins. Besides above-mentioned, the disease annotations from Ingenuity Pathway Analysis (IPA) were as the references to provide the disease linkage from human, mouse and rat. Moreover, the expression localization in tissues and cell lines and the biomarker filtering of S-nitrosylated proteins was also referred to assign the known or unknown markers in diseases. In addition, the endogenous *S*-nitrosoproteomic data set from 11 pairs of individualized tissues of CRC patients was also integrated (44). This study not only provides the first *S*-nitrosoproteomic analysis in human tissue but also enables a better understanding of the effect of endogenous SNO in cancer.

### Reconstruction of protein SNO regulatory network using metabolic pathways and PPIs

An increasing number of studies suggested that protein SNO plays a crucial role in the regulation of NO signaling pathway (45–49). Thus, one of the aims in this update is integrating the information of metabolic pathways and PPIs to reconstruct the SNO regulatory network for a group of interested genes/proteins. The information of metabolic pathways was referred to the annotations in KEGG (50). For the information of experimentally verified physical interactions, over 10 PPI databases (as listed in Supplementary Table S1) have been integrated into dbSNO. As presented in Supplementary Figure S3, given a group of interested proteins, the proteins were searched for *S*-nitrosylated annotations in dbSNO and mapped on metabolic pathways by Cytoscape program (51). Additionally, the PPIs associated with the interested proteins were provided to discover new members that have the potential for involving in a mapped metabolic pathway. In order to make the reconstruction of SNO networks feasible, a graph theory (as described in Supplementary Figure S3) has been adopted to formalize the networks based on a KEGG pathway map.

## DATA CONTENT AND UTILITY

### Data statistics and amino acid composition for SNO sites

After manually reviewing over 500 research articles obtained from a text mining method, up to September 1, 2014, a total of 276 SNO*-*associated articles covering 18 organisms are retrieved from PubMed. In this update, after removing redundant data among these heterogeneous resources, 4165 SNO cysteines on 2277 SNO proteins are presented. According to the classification of experiment methods, 1169 SNO sites on 768 proteins were assigned *in vivo* and 2076 SNO sites on 1195 proteins are assigned *in vitro*. For the *in vivo* experiment, totally 575 SNO sites on 376 proteins and 577 SNO sites on 378 proteins are identified from human and mouse, respectively. As for *in vitr*o experiments, 273 SNO sites on 206 proteins and 1641 SNO sites on 873 proteins are identified from human and mouse, respectively.

**Figure 1.**
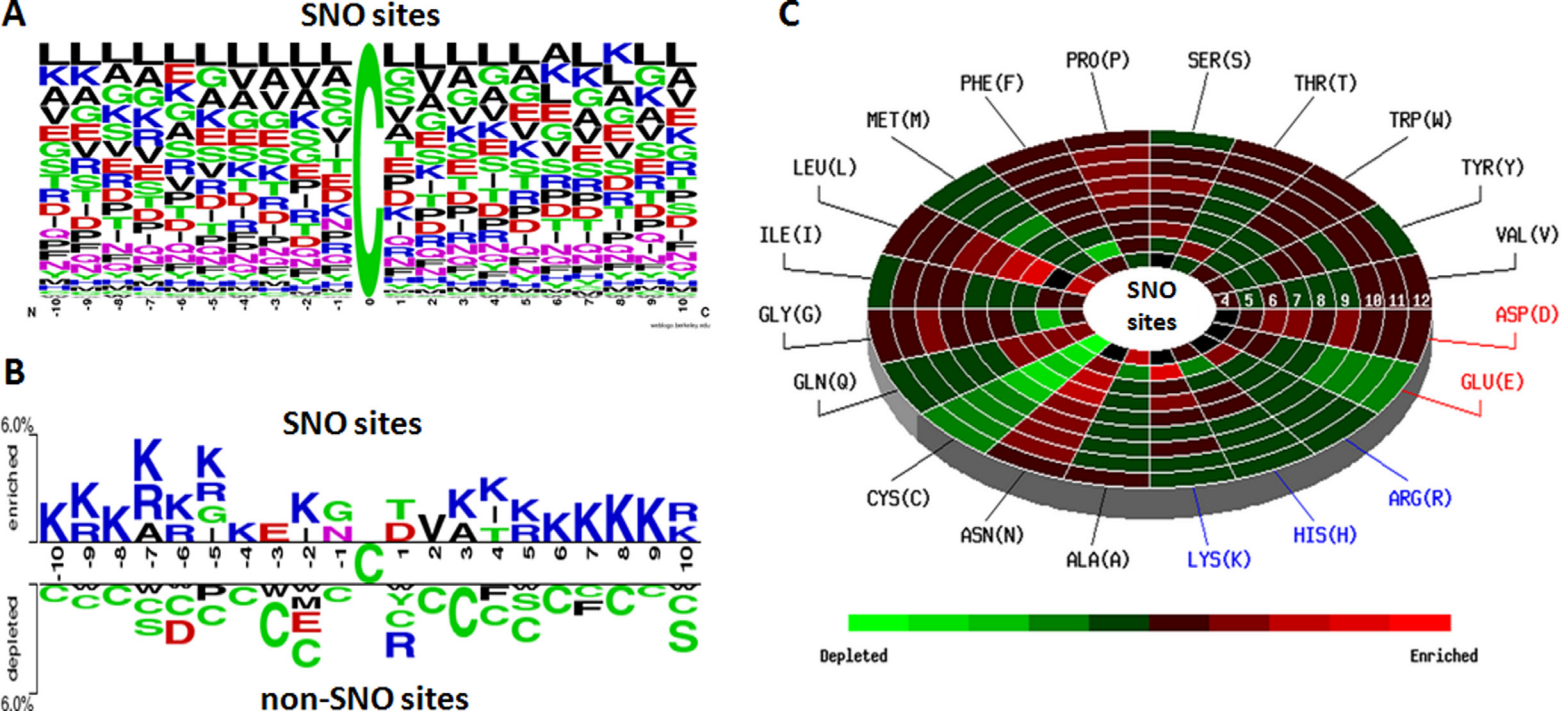
Composition of amino acids surrounding *S*-nitrosylation sites. (**A**) Position-specific amino acid composition surrounding SNO sites. (**B**) Comparison of position-specific amino acid composition between SNO sites (top) and non-SNO sites (bottom). (**C**) A radial cumulative propensity plot of spatial amino acid composition surrounding the SNO substrate sites of protein tertiary structures.

In the investigation of substrate site specificity, Figure 1A shows that there is no significant motif surrounding the SNO sites. However, the Two Sample Logo (52), a comparison of position-specific amino acid composition between SNO sites (top) and non-SNO sites (bottom), shows that the positively charged Lysine (K) and Arginine (R) residues are highly abundant around SNO sites, as presented in Figure 1B. The data statistics, position-specific amino acid composition and the Two Sample Logo for the SNO sites in each organism are presented in Supplementary Table S2. The positively charged residues (K and R) are also enriched around human and mouse SNO sites. With the difficulty to identify the conserved motifs from a large-scale data set, MDDLogo (53) has been applied to human and mouse SNO peptides for the detection of substrate motifs. As shown in Supplementary Tables S3 and S4, totally 11 and 12 substrate motifs, which contain the conserved motifs of positively charged amino acids (K, R and H) at a specific position, are identified from 1250 human and 2647 mouse SNO sites, respectively, using a 21-mer sequence length. Consistent with previous studies (12,14,54), the SNO cysteines have the preference to locate in the regions flanking with acidic and basic amino acids.

Figure 1C shows the radial cumulative propensity plot (spatial neighborhood) of amino acid composition surrounding 298 SNO substrate sites based on tertiary structures. According to the observation from Two Sample Logo, SNO has the significant enrichments of K and R residues in the sequential neighborhood of substrate sites. However, the radial cumulative propensity plots present that there is an additional enrichment of amino acids in the spatial neighborhood. In addition to the K and D residues, there exists an enrichment of hydrophilic residues, Asparagine (N) and Proline (P), accompanied by a remarkable depletion of hydrophobic Cysteine (C) residue in the spatial neighborhood.

### Enhanced web interface for structural investigation of SNO substrate sites

**Figure 2.**
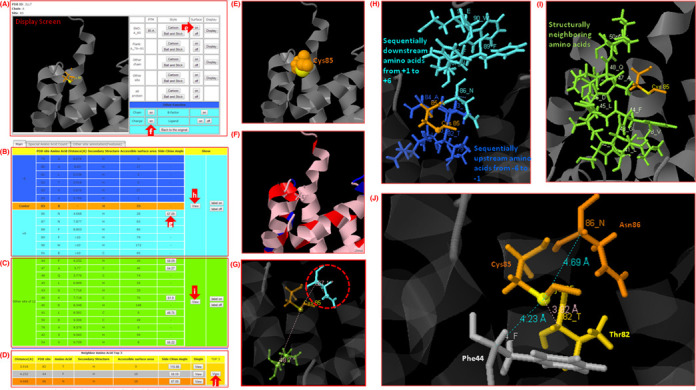
A case study for the spatial context of an *S*-nitrosylation substrate site of cysteine (Cys85) in the protein tertiary structure (PDB ID: 2LLT) of protein S100-A1 (UniProtKB ID: S10A1_HUMAN). (**A**) Structural overview. (**B**) Table of sequentially neighboring amino acids. (**C**) Table of structurally neighboring amino acids. (**D**) Table of top three nearest amino acids. (**E**) Surface area view. (**F**) The acid residues (blue) and basic residues (red) of SNO substrate site. (**G**) Side chain orientation of neighboring amino acids. (**H**) Structural view of sequentially neighboring amino acids. (**I**) Structural view of structurally neighboring amino acids. (**J**) Detailed view of top three nearest amino acids.

To enhance the utility of dbSNO resource, the web interface has been improved for users to browse and search efficiently for their proteins of interest. Supplementary Figure S4 shows the data content of a typical dbSNO entry. One of the aims in this update is to provide a platform for structural investigation of SNO substrate sites based on protein tertiary structures of PDB. Figure 2A presents a case study for investigating the spatial context around an SNO substrate site of Cys85 in the tertiary structure (PDB ID: 2LLT) of Protein S100-A1 (UniProtKB ID: S10A1_HUMAN). The tables of sequentially and structurally neighboring amino acids with side chain orientations are provided in Figure 2A and C, respectively. The table of top three nearest amino acids structurally neighboring the SNO substrate site of Cys85 is provided in Figure 2D. The surface area of Cys85 is also given in Figure 2E for the analysis of solvent accessibility. In order to provide a full investigation of structural acid-based motif (55) surrounding the SNO substrate site, the acid residues (K, R and H) and basic residues (D and E) are marked in blue and red, respectively, as shown in Figure 2F. The sequentially upstream (from positions −6 to −1) and downstream (from +1 to +6) amino acids, in which Asn86 (at position +1) contains the side chain orientation of 67.09° (as shown in Figure 2G) to the thiol group (SG) of Cys85 on the protein structure, are provided in Figure 2H. Figure 2I shows that the structurally neighboring amino acids, whose radial distance to the thiol group of Cys85 is less than 10 Å, are marked with highlighted blue on protein 3D structure. Furthermore, in the investigation of structurally neighboring amino acids, the top three nearest amino acids (Thr82, Asn86 and Phe44) with the information of accessible surface area and side chain orientation are provided in Figure 2J. Interestingly, Phe44 residue, which is distant to Cys85 in linear sequence, is accessible to protein surface area and contains the side chain orientation of 58.59°. This investigation indicates that Phe44 residue may provide significant influence on the binding of NO to Cys85. Additionally, five cases for local and global structure alignment between protein containing S-nitrosylated cysteine (SNC) and protein containing cysteine without NO binding are presented in Supplementary Figure S5.

### Functional associations of *S*-nitrosylated proteins

**Figure 3.**
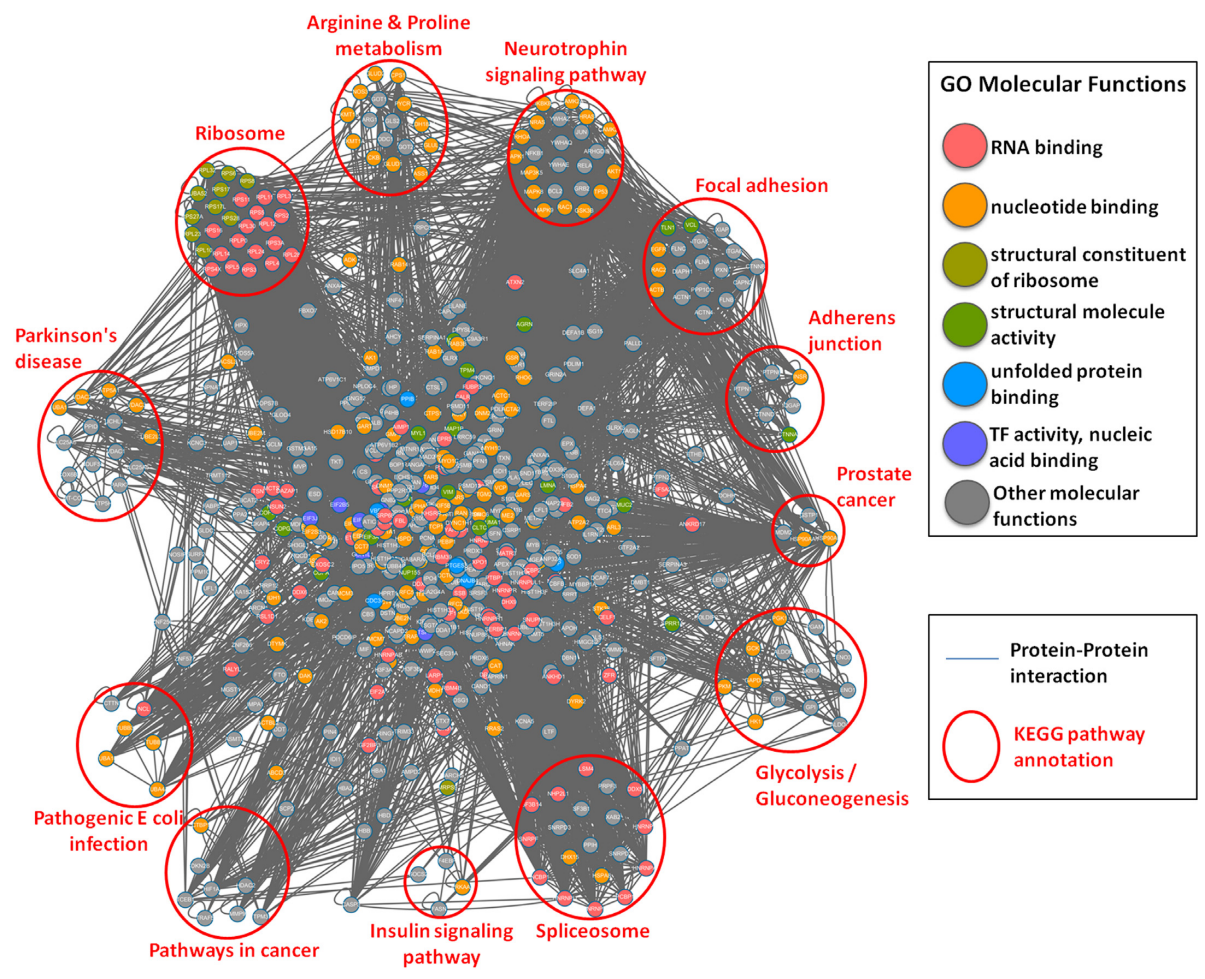
Protein–protein interaction network of all human *S*-nitrosylated proteins with the annotations of GO molecular function (marked in different colors) and KEGG metabolic pathways (clustered in different pathway groups).

Based on the annotations of GO database, the distributions of the biological process, molecular function and cellular component for human and mouse general and SNO proteins are presented in Supplementary Tables S5–S8, respectively. According to the annotations of KEGG pathways, the distributions of pathway annotations for human and mouse SNO proteins are provided in Supplementary Tables S9–S12, respectively. Figure 3 provides a global network view for PPI of all 720 human SNO proteins with the annotations of GO molecular function and KEGG metabolic pathways. The result could provide a preview of the SNO protein family clustering involving in what major functional annotation and biological pathway, such as RNA binding family in ribosome and spliceosome, structural molecule activity family in focal adhesion and adherens junction and enzymes in glycolysis/gluconeogenesis, cancer and disease regulation.

According to the annotations of InterPro database, ∼65% of the reported SNO sites are located within functional domains. Compared to total cysteine residues on SNO proteins, ∼38.6% of total cysteine residues were located on functional domains, indicating SNO may provide functional roles in various biological processes. Supplementary Tables S13–S16 provide the distributions of top 20 InterPro functional domains covering total cysteine residues and SNO sites in human and mouse, respectively. This investigation shows that many SNO sites prefer to locate within nucleotide (DNA or RNA) binding domain and NAD(P)-binding domain.

### Disease associations of human *S*-nitrosylated proteins

**Figure 4.**
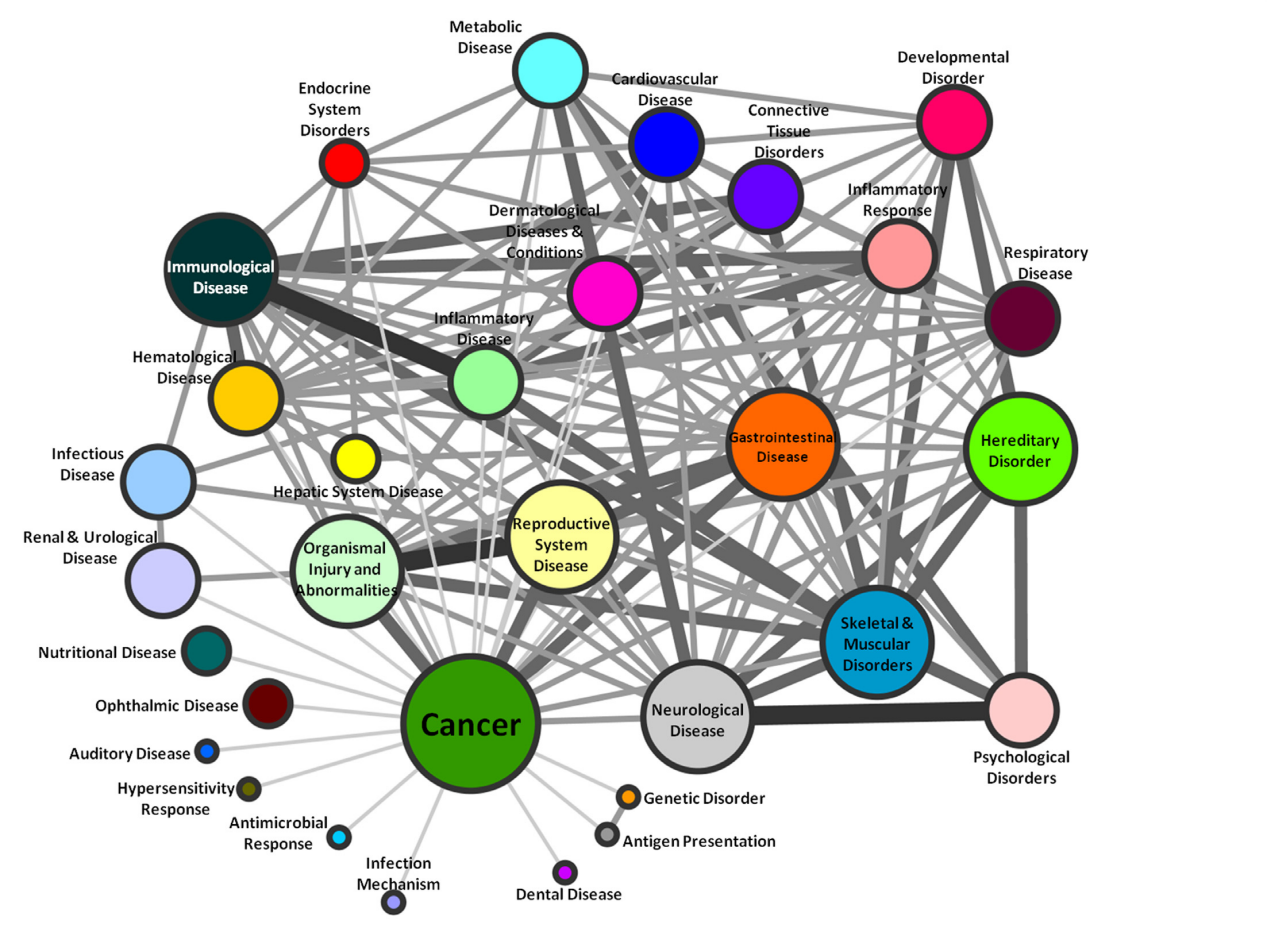
Disease network for human *S*-nitrosylated proteins.

Based on the information from OMIM, KEGG and IPA database analysis, the correlation of these 720 human SNO proteins with disease-related processes and networks was also analyzed and categorized into 31 diseases. As shown in Figure 4, 370 (51%) of SNO proteins were matched to cancers, including oral, breast, gastric, colorectal, liver cancer, etc. (Supplementary Table S17). In addition to cancer, other top four diseases were described as organismal injury and abnormalities, reproductive system disease, gastrointestinal disease and neurological disease. The correlations of diseases linking with their common proteins were presented to clarify the relationship of SNO proteins and diseases. Taken together, the results showed that these SNO proteins display annotated roles in the disease regulation. The connecting information of these diseases can help users to understand whether the SNO of these proteins plays the same or different roles in correlated diseases. Most importantly, the relationship between different diseases for SNO may supply the new respect to find out the potential regulators that modulate the disease initiation, progression or suppression.

### Reconstructing SNO regulatory (NO signaling) network for a group of interested proteins

Supplementary Figure S6 presents the web interface of reconstructing protein SNO network considering both the information of metabolic pathways and PPIs. In network visualization, the queried proteins that can be mapped to a specific member of a metabolic pathway are represented as squares colored with light blue. The queried proteins that could not be mapped to a specific member of a metabolic pathway are represented by blue circles. Moreover, the PPIs associated with the queried proteins are displayed as yellow lines. To further explore the relationship and functional assignments in disease and signaling pathway of SNO, the *S*-nitrosoproteomic networks are assembled through reported direct and indirect PPIs and the human disease networks using OMIM and KEGG pathway. For instance, the CRC pathway from KEGG database is used as a case study. As shown in Supplementary Figure S7, 13 proteins and 11 SNO sites are matched in the classical CRC pathway based on the dbSNO data set. To further explore NOS2 and TXN, two well-studied regulating enzymes, in CRC, their associated proteins are also presented in CRC pathway by PPIs. Given a group of interacting proteins of one SNO target protein, its regulating upstream and downstream targets are also provided to discover new members that have the potential for involving in a mapped pathway. Taken together, the result provides us with a potential regulatory network connecting with the classical pathway that NOS2- and TXN-regulated protein family and their *S*-/*trans*-/de-nitrosylation function in cancer.

## CONCLUSION

Due to the emerging roles of SNO proteins in the development and progression of cancers and diseases, dbSNO is updated as a resource for full investigation of large-scale *S*-nitrosoproteome. The improvements and advances in dbSNO 2.0 are summarized in Table 1. Due to a variety of site-specific identification methods, the SNO targets and substrate sites are further defined as *in vivo* or *in vitro* according to their experimental models. The increasing interest in the structural environment of PTM sites prompted dbSNO to design a platform for providing the information of spatial amino acid composition, solvent-accessible surface area, spatially neighboring amino acids and side chain orientation for 298 SNO substrate sites based on protein tertiary structures. Owing to the importance of protein SNO in regulating protein functions and pathophysiological processes in various human disorders, the annotations of protein molecular functions, biological processes, functional domains and human diseases are integrated to explore the functional and disease associations for SNO proteins. Most importantly, the *S*-nitrosoproteomic study has been fueled by advances of high-throughput MS-based proteomic technologies that provide researchers with the updated dbSNO for exploring NO signaling dynamics.

**Table 1.** The improvements and advances in dbSNO 2.0 (September 1, 2014)

	dbSNO 1.0	dbSNO 2.0
Number of organisms	18	18
Number of cysteine *S*-nitrosylation instances	3374	4165
Number of *S*-nitrosylated proteins	1757	2277
Number of supported literatures	219	276
Number of *in vivo* SNO sites/proteins	-	1169 sites/768 proteins
Number of *in vitro* SNO sites/proteins	-	2076 sites/1195 proteins
Substrate motif analysis	WebLogo	WebLogo, MDDLogo and TwoSampleLogo
Functional domain	-	InterPro
Structural characteristics of SNO sites	-	Spatial amino acid composition, solvent-accessible surface area, spatially neighboring amino acids and side chain orientation
Protein–protein interaction	-	Over 10 public PPI resources
Network analysis of SNO proteins	-	Network analysis with protein–protein interactions, KEGG and human disease database
Network visualization	-	PHP GD library and Cytoscape package
Disease information	-	KEGG Disease database, OMIM, The Human Protein Atlas
Correlation between SNO and other types of PTM	-	Correlation network between SNO and other 13 PTM types

In the future, the growth of dbSNO is expected as the availability of data increases in research articles related to protein SNO, especially for the *S*-nitrosoproteome data involved in human diseases. To provide more adequate information for functional and disease analysis, the descriptions associated with the biological function of SNO sites will be extracted with increased precision from full-text articles by using an enhanced information retrieval system. The SNO site-specific information associated with biological functions or disease annotations will greatly help the study of protein SNO in prospective works. Another area that we can envision dbSNO improving significantly in the future is in having the survey of SNO near drug binding sites in the protein tertiary structures and their effects (56).

## AVAILABILITY

The data content in dbSNO 2.0 will be maintained and updated quarterly by continuously surveying the public resources and research articles. Also, the *S*-nitrosoproteome data involved in human diseases will be semiannually updated by manually reviewing the research articles. The updated resource is now freely accessed online at http://dbSNO.mbc.nctu.edu.tw/. All of the experimentally verified SNO sites could be downloaded in the text format.

## SUPPLEMENTARY DATA

Supplementary Data are available at NAR Online.
